# Dietary protein intake and overall diet quality in adults with cystic fibrosis following elexacaftor/tezacaftor/ivacaftor therapy

**DOI:** 10.1017/S0007114525103760

**Published:** 2025-06-28

**Authors:** Paul T. Morgan, Tanith-Jade Ellis, Benoit Smeuninx, Leigh Breen, Laura Kinsey, Owen W. Tomlinson, Helen White, Laura R. Caley, Daniel G. Peckham

**Affiliations:** 1 Department of Sport and Exercise Sciences, Institute of Sport, Manchester Metropolitan University, 99 Oxford Road, Manchester, UK; 2 Monash Institute of Pharmacological Sciences, Monash University, Parkville, VIC, Australia; 3 School of Sport, Exercise and Rehabilitation Sciences, University of Birmingham, Edgbaston, Birmingham, UK; 4 Manchester Adult Cystic Fibrosis Centre, Wythenshawe Hospital, Manchester University Hospitals NHS Foundation Trust, Manchester, UK; 5 University of Exeter Medical School, St Luke’s Campus, Exeter, UK; 6 Nutrition, Health & Environment, Leeds Beckett University, Leeds, UK; 7 Leeds Institute of Medical Research, University of Leeds, Leeds, UK; 8 Department of Respiratory Medicine, Leeds Teaching Hospitals NHS Trust, Leeds, UK

**Keywords:** Ageing, CFTR Modulators, Cystic Fibrosis, Dietary Protein, Diet Quality

## Abstract

The RDA for dietary protein is likely insufficient for individuals with cystic fibrosis (CF). This study sought to characterise protein intake and diet quality in adults with cystic fibrosis (awCF), before and after elexacaftor/tezacaftor/ivacaftor (ETI) therapy, compared with healthy controls. Dietary intake was assessed by diet diary in awCF at baseline (BL, *n* 40) and at follow-up > 3 months post ETI therapy (follow-up (FUP), *n* 40) and in age-matched healthy controls (CON, *n* 80) free from known disease at a single time point. Protein intake dose and daily distribution, protein quality, protein source and overall diet quality were calculated for each participant. Both CON (1·39 (sd 0·47) g·kg^–1^·day^–1^) and CF (BL: 1·44 (sd 0·52) g·kg^–1^·day^–1^, FUP: 1·12 (sd 0·32) g·kg^–1^·day^–1^) had a higher mean daily protein intake than the protein RDA of 0·75g·kg^–1^·day^–1^. There was a significant reduction in daily protein intake in the CF group at FUP (*P* = 0·0003, *d* = 0·73), with levels below the alternative suggested dietary intake of ≥ 1·2 g·kg^–1^·day^–1^. There were no sex differences or noticeable effects on protein quality or source following the commencement of ETI therapy when compared with CON (all *P* > 0·05), although overall diet quality decreased between time points (*P =* 0·027, *d* = 0·57). The observed reduction in daily protein intake in the present cohort emphasises the importance of ensuring appropriate dietary protein intake to promote healthy ageing in adults with CF. More research is needed to evidence base dietary protein requirements in this at-risk population.

Current estimates suggest ∼150 000 people worldwide are diagnosed with cystic fibrosis (CF)^(1)^. CF is an autosomal recessive disorder caused by mutations in the CF transmembrane conductance regulator (CFTR) gene. This results in the absence or dysfunction of the CFTR protein, a protein which functions as an anion channel, conducting chloride and bicarbonate, and regulating sodium transport. The ionic imbalance in CF is associated with dehydrated and acidic airway surface liquid, a predisposition to lung infections, innate inflammation, and tenacious secretions in both respiratory and digestive systems^(2)^. Aberrations in normal physiology also impair pulmonary and digestive function, nutrient absorption, and predispose individuals to diabetes, osteoporosis, liver disease, colorectal cancer and skeletal muscle dysfunction^(2–5)^. Historically, treatment for CF has been based on frequent and intense antibiotic therapies, airway clearance, exercise and nutritional regimens, which often included a high fat and low fibre diet^(6–9)^. However, this emphasis on a high calorie intake at any cost has recently changed with the introduction of CFTR modulators; a new class of drug targeting the underlying defect rather than disease complications. Elexacaftor/tezacaftor/ivacaftor (ETI) is the newest combination to be licensed, being effective for ∼85 % of people with CF (pwCF)^(10)^. Treatment with ETI results in improved quality of life, lung function and weight gain, as well as reduced exacerbations, even in those with advanced pulmonary disease^(11–15)^. Alongside significant improvements in quality of life, pwCF who are treated with highly effective modulator therapy have a projected median survival of > 71 years, with children born today having a relatively normal life expectancy^(16)^. Indeed, in 2014 life expectancy for pwCF was as low as 40 years^(17)^. These transformative changes are leading to an ageing CF population. A hallmark of non-CF ageing is progressive and accelerated loss of skeletal muscle mass, quality, and function, termed sarcopenia^(18)^. These changes contribute to loss of functional health and increased morbidity, highlighting the importance of maintaining skeletal muscle health in ageing^(18)^. Reduced muscle mass and function is also present in pwCF, reflecting a complex milieu of malnutrition, infection, inflammation and dysregulated calcium homeostasis in skeletal muscle^(19,20)^. Importantly the reported increase in weight, post ETI, may be more reflective of alterations in the fat compartment rather than muscle mass^(15,21)^. Reduced muscle mass, quality, and function are also independently associated with disease progression^(5,22,23)^, but until now, counteracting symptoms of accelerated ageing has not been a clinical priority in pwCF.

The adequate supply of essential amino acids (EAA) is necessary for a positive protein balance, the stimulation of muscle protein synthesis (MPS), and the prevention of skeletal muscle mass loss^(24)^, which pwCF are at risk of^(25)^. In the postprandial period, dietary protein robustly stimulates MPS contributing to net muscle protein accretion^(26)^. However, an impaired muscle anabolic response to the ingestion of lower doses of protein in older individuals, termed skeletal muscle ‘anabolic resistance’, is thought to be a critical factor in age-related muscle deterioration^(27)^. In addition, the recommended dietary allowance (RDA) for protein (UK: 0·75 g·kg^−1^·day^−1^) is thought to be insufficient for repeated, robust stimulation of MPS and hence maintenance of muscle in older adults without CF, the critically ill and those with chronic respiratory disease^(28,29)^. Indeed, higher protein intakes of > 1·2 g·kg^−1^·day^−1^ are associated with increased muscle mass, quality and function in older individuals^(29–32)^. In addition to the dose, the quality of dietary protein is an important determinant of postprandial MPS stimulation and skeletal muscle remodelling. Protein quality is defined by a number of factors including the EAA content, profile and bioavailability, combined with protein and/or amino acid (AA) needs, and the digestion kinetics and delivery of AA to biological tissues for protein synthesis^(29)^. In a typical Western diet, protein consumption primarily (>50%) originates from animal products, which have an EAA profile closely matching bodily requirements^(29,33,34)^. Notwithstanding, there have been calls to increase the intake of plant-based proteins, in part owed to increased health, environmental and ethical concerns associated with animal-based foods^(35)^. This is despite significant impairments in nutrient absorption and reduced muscle mass, quality and function in pwCF^(5,22,23,36)^. We envisage that an ageing CF population will be at a greater risk for sarcopenia due to persistent infections, inflammation, gastrointestinal abnormalities and a catabolic state. Therefore, combined with the anticipated improvements in lifespan, a better understanding of dietary protein intake in CF is urgently required.

In the current study, our primary aim was to comprehensively characterise protein intake and overall diet quality in this population of adults with cystic fibrosis (awCF) pre- and post-ETI therapy, comparing against current UK recommendations for non-CF adults and a healthy control group free from known disease.

## Methods

### Study design and ethical approval

A portion of the data presented herein have previously been published elsewhere^(15,37)^. Briefly, this study was part of a prospective observational cohort project conducted across four UK Adult CF Care Centres (Leeds, Royal Papworth, Birmingham, Manchester)^(15)^, with control participants being recruited as part of a separate study in Birmingham, UK^(37)^. A total of forty pancreatic insufficient awCF were recruited for this part of the CF cohort study (age at baseline (BL): 35·6 (sd 9·8) years; BMI at BL: 23·3 (sd 2·8) kg·m^–2^). At BL, 15 awCF (38 %) were on double therapy, which is less clinically effective than ETI, and not associated with such significant changes in BMI^(15)^. Exclusion criteria for CF participants comprised lung transplant recipients, prognosis < 6 months, pregnant or having another significant gastrointestinal pathology. Favourable ethical opinion was received from London Bromley Research Ethics Committee (REF: 18/LO/2241). Healthy controls (CON) were age-matched to the CF group (*n* 80; age: 37·7 (sd 14·6) years; BMI: 25·0 (sd 5·0) kg·m^–2^) and eligible if free from disease and deemed ostensibly healthy based on a general health questionnaire. Control participants were recruited from the Birmingham area (West Midlands, UK), and ethical approval was obtained through the University of Birmingham Research Ethics Committee (REF: 13–1475A). Finally, this study was approved by the Science and Engineering Ethics Committee of Manchester Metropolitan University (Ref No. EthOS 52086). Across the 120 participants, both sexes were recruited near-evenly (male: 52 %, female 48 %). Voluntary, written, informed consent was received from all CF and CON participants. This study was conducted according to the guidelines laid down in the Declaration of Helsinki.

### Dietary data collection

For the CF group, the study had two time points: BL, and follow-up (FUP), originally scheduled 6 months apart as part of a wider study^(15)^. However, the study paused from March until December 2020 owing to the COVID-19 pandemic. The pause resulted in a > 6-month gap between time points for those awCF partway through data collection. During this time, ETI modulator therapy became more widely available in the UK, being licensed in mid-2020^(38)^. This significant clinical development was incorporated into the CF observational study with FUP data being collected ≥ 3 months after commencing ETI therapy for these individuals. For participants with CF, the most recent clinical weight measurement was recorded for each time point. For the healthy control group, dietary intake was assessed at a single time point. All participants recorded all food, fluid and any oral nutritional supplements or enteral nutrition for 3–4 d (two or three weekdays and one weekend day) by diet diary. For more detailed information on the study design(s), including details relating to dietary recording, see Caley *et al.* (2023)^(15)^ and Smeuninx *et al.* (2020)^(37)^.

### Analytical methods: calculating protein intake and diet quality

Initially, daily mean nutritional intake at each time point was calculated for each participant. Thereafter, protein intake dose (relative to bodyweight in kilograms), protein intake distribution throughout the day (relative to bodyweight in kilograms), protein quality, relative (%) dietary protein source intake and overall diet quality were calculated for each participant.

Protein intake dose was calculated by dividing total daily protein intake by the corresponding participant’s bodyweight in kilograms, with improved relevance for recommendations for skeletal muscle anabolism. Relative protein intakes were also compared with the current RDA for dietary protein consumption in the UK of 0·75 g·kg^−1^·day^−1^. Similarly, protein intake distribution (or ‘meal-specific protein intake’) was calculated by dividing total daily protein intake at each meal opportunity by the participant’s corresponding bodyweight and separated into breakfast, lunch, dinner and snacks. Relative protein intake at each meal was compared against 0·24 g·kg^−1^ and 0·40 g·kg^−1^ thresholds for maximal MPS stimulation for young (based on 18–35-years-olds) and older (based on > 60-year-olds) individuals, respectively, and used to assess the proportion of meals that reached these respective thresholds^(39)^. The number of individuals reaching the RDA for protein of 0·75 g·kg^−1^·day^−1^ and higher 1·2 g·kg^−1^·day^−1^ recommendation within each group (based on the notion of 3 × 0·40 g·kg^−1^ and following recent calls for increases in the current protein RDA^(40)^) were expressed as a percentage of the total group.

To compare protein quality across groups and time points, we multiplied the protein dose by the corresponding Digestible Indispensable AA Score as reported by Adhikari *et al.* (2022)^(41)^, incorporating ileal digestibility, to provide a score that reflected a combination of the quality and total intake (in grams) of protein, whereby a higher score reflects higher quality. Whilst we acknowledge that the Digestible Indispensable AA Score might be considered a somewhat crude assessment of protein quality for human nutrition purposes^(42)^, there are no existing universally agreed means to quantitatively assess dietary protein quality that would address the limitations of the current systems that are available and the Digestible Indispensable AA Score represents the most accurate means to routinely give a single protein quality value for a stand-alone food^(42)^. Therefore, as an additional marker of protein quality, we also calculated protein intake relative to total caloric intake. The relative intake of dietary protein sources was presented by separating protein foods by the following categories: (a) Meat & Poultry; (b) Fish; (c) Dairy/Eggs; (d) Cereal, Grain & Bread; (e) Fruit/Vegetable; (f) Other Animal; (g) Other Plant; (h) Other, and expressed as a percentage of total protein intake. Finally, overall diet quality was assessed according to the Healthy Eating Index, whereby a higher value (out of 100) corresponds to higher dietary quality based on the consumption of the following food groups: Total Fruits, Whole Fruits, Total Vegetables, Greens and Beans, Whole Grains, Dairy, Total Protein Foods, Seafood & Plant Proteins, Fatty Acids^(43)^. In addition, the Heating Eating Index accounts for moderation of the following: Refined Grains, Sodium, Added Sugars, Saturated Fats^(43)^.

### Primary outcome measures

For analysis, the primary outcomes were daily protein intake dose, protein intake distribution throughout the day (i.e. meal-specific protein intakes), protein quality and overall diet quality. For comparative purposes, the aims of this study were threefold, to assess: (1) the change in protein intake and diet quality in awCF following initiation of ETI; (2) the differences in protein intake and diet quality between awCF not on ETI modulator therapy and a control healthy population and (3) the differences in protein intake and diet quality between awCF following initiation of ETI modulator therapy and a control healthy population.

### Statistical analyses

Paired *t* tests were used to compare the effects of ETI modulator therapy on daily protein intake, protein quality and overall diet quality in CF (i.e. BL compared with FUP). Independent *t* tests were employed to assess differences between CF at BL and CON and differences between CF at follow-up (i.e. FUP) and CON in daily protein dose, protein quality and overall diet quality. Mixed model ANOVA were employed to assess the effects of ETI therapy on protein distribution throughout the day, as well as differences between CF at BL and CON, and between CF at follow-up (i.e. FUP) and CON in protein distribution. Where the ANOVA revealed a significant effect, post hoc analysis was conducted, using a Bonferroni correction, to isolate specific between-group differences. For all tests, to assess any differences in or influence of sex, analyses were repeated, separating by biological sex at birth (male, female). Cohen’s *d* was used to calculate the effect size for *t* tests and post-hoc comparisons, where *d* = 0·2, 0·5 and 0·8 indicate small, medium and large effects, respectively. Where sphericity was violated, a Greenhouse–Geisser correction factor was used. Normal distribution was assessed using the Shapiro–Wilk test. Where appropriate, non-normally distributed variables were logarithmically transformed. For all tests, results were considered statistically significant when *P* < 0·05. Data are presented as mean (standard deviation) or standard error of the mean, unless otherwise indicated. All statistical analyses were conducted using IBM SPSS Statistics version 28.

## Results

For more detailed demographics, clinical characteristics and macronutrient intake of CF participants, see Caley *et al.* (2023)^(15)^. More detailed information from CON can be seen in Smeuninx *et al.* (2020)^(37)^. For context of disease severity, in the CF cohort, average ppFEV1 (percentage predicted forced expiratory volume in one second) was 46·8 % (interquartile range 34·8–65·8) at BL and 56·5 % (interquartile range 43·5–72·6) at FUP. CF-related diabetes and CF-related liver disease were diagnosed in 38 % and 30 % (¼ of whom had cirrhosis of the liver) of the cohort, respectively.

### Daily protein intake dose

**Figure 1.**
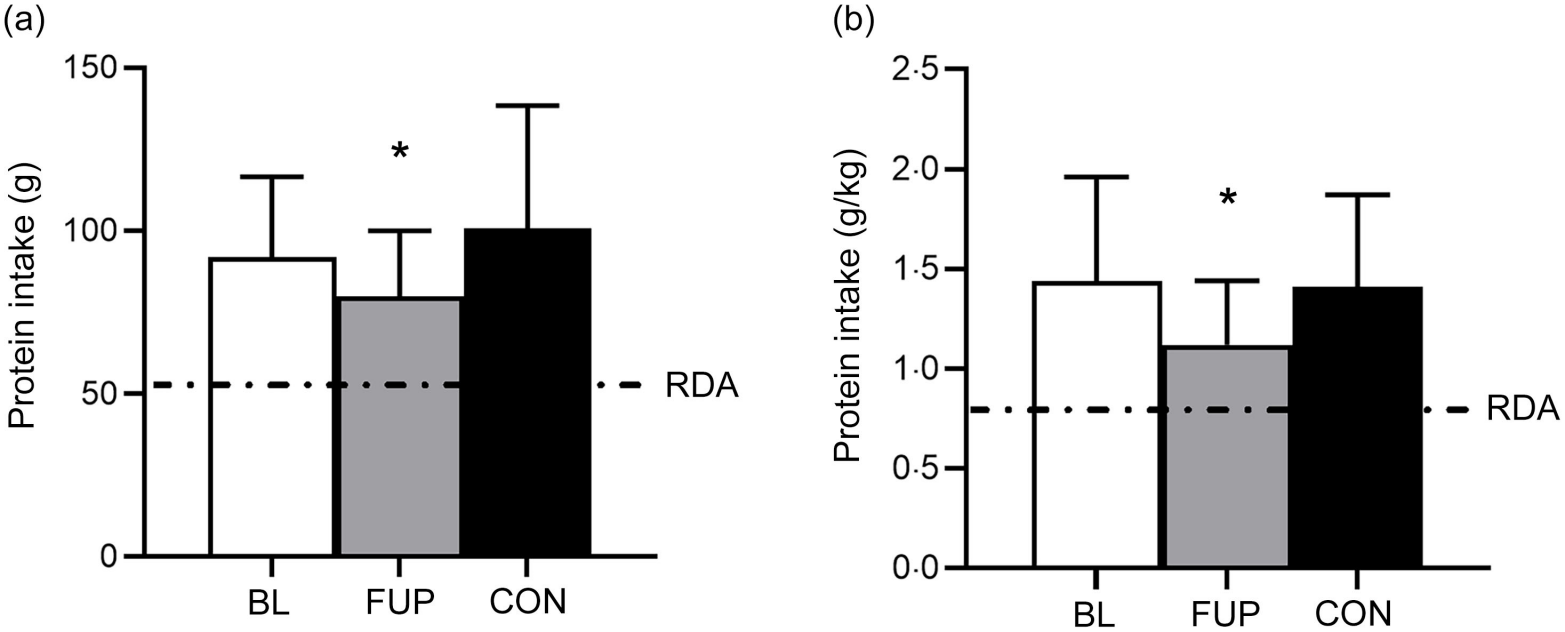
Daily protein intake for baseline (BL, clear bar), follow-up (FUP, grey bar) and healthy controls (CON, black bar) are shown in Panel A. Daily protein intake relative to bodyweight (in kilograms) is shown in Panel B. The dashed line represents the current RDA for protein in the UK (0·75 g·kg^–1^·day^–1^). The dashed line in Panel A represents a typical 70 kg individual. Values are presented as means (sd). Significance was set at *P* < 0·05. * Significantly different to BL and CON.

An overview of daily dietary protein intake for CF at BL and FUP and for CON can be viewed in Fig. 1 and Table 1. Average daily protein intake was above the protein RDA of 0·75 g·kg^–1^·day^–1^ for all groups and at all time points (Fig. 1). There was no difference between daily protein intakes in the CF group at BL compared with CON (BL: 1·44 (sd 0·52) g·kg^–1^·day^–1^; CON: 1·39 (sd 0·47) g·kg^–1^·day^–1^, *P* = 0·63, *d* = 0·09, Fig. 1). However, daily protein intakes were 28 % and 24 % lower at FUP (1·12 (sd 0·32) g·kg^–1^·day^–1^) compared with BL (*P* = 0·0003, *d* = 0·73) and CON (*P* = 0·001, *d* = 0·67), respectively. There were no differences in relative protein intake (i.e. % contribution of total caloric intake) between groups or any effects of sex on daily protein intake (all *P* > 0·05, Table 1).

93 % and 98 % of participants met the current RDA for protein intake of 0·75 g·kg^–1^·day^–1^ for awCF (at BL) and CON, respectively, which reduced to 90 % in CF at FUP. However, only 72 % of CF participants at BL and 64 % of CON group met the RDA on all measurement days. In CF participants at FUP, this reduced to 55 % meeting the RDA on all recorded measurement days. When compared to the alternative higher protein recommendation of 1·2 g·kg^–1^·day^–1^, a greater proportion of CON (60 %) reached this protein intake on all three individual measurement days compared with the CF group both at BL (30 %) and FUP with a further reduction (10 %).

**Table 1.** Comprehensive summary of daily dietary protein intake for CF at BL and CF at FUP and for CON

	CF at BL (*n* 40)		CF at FUP (*n* 40)		CON (*n* 80)	
	Mean	sd	Mean	sd	Mean	sd
Total daily protein intake (g·day^–1^)	92·0	24·6	79·9	20·1^*,†^	100·6	37·8
Male	97·7	24·9	85·6	20·2^*,†^	115·2	42·8
Female	87·8	23·8	71·4	17·6^*,†^	87·3	26·8
Total daily protein intake (g·kg^–1^·day^–1^)	1·44	0·52	1·12	0·32^*,†^	1·41	0·46
Male	1·36	0·47	1·10	0·27^*,†^	1·48	0·51
Female	1·53	0·57	1·14	0·37^*,†^	1·35	0·42
Meal-specific protein intake						
Breakfast protein intake (g)	16·5	10·5	14·2	7·4	15·0	10·1
Male	15·3	6·6	15·3	8·7	17·9	12·1
Female	17·8	13·6	13·0	5·6	12·4	7·1
Lunch protein intake (g)	27·0	10·9	20·8	9·2^*,†^	30·8	16·9
Male	29·3	9·5	24·1	10·1^*,†^	37·5	21·0
Female	24·5	12·1	17·2	6·5^*,†^	24·7	8·7
Dinner protein intake (g)	38·5	15·2	35·5	11·8	42·0	17·8
Male	40·9	17·1	35·9	12·4	43·4	18·6
Female	35·8	12·6	35·0	11·4	40·7	17·2
Snacks protein intake (g)	11·0	8·1	8·4	7·2	12·8	14·0
Male	12·2	8·4	10·3	7·4	16·3	16·6
Female	9·6	7·9	6·2	6·5	9·5	10·3

CF, cystic fibrosis; BL, baseline; FUP, follow-up; CON, control. *Denotes significant difference from CON (*P* < 0·05). ^†^Denotes significant difference from BL (*P* < 0·05).

### Dietary protein distribution

**Figure 2.**
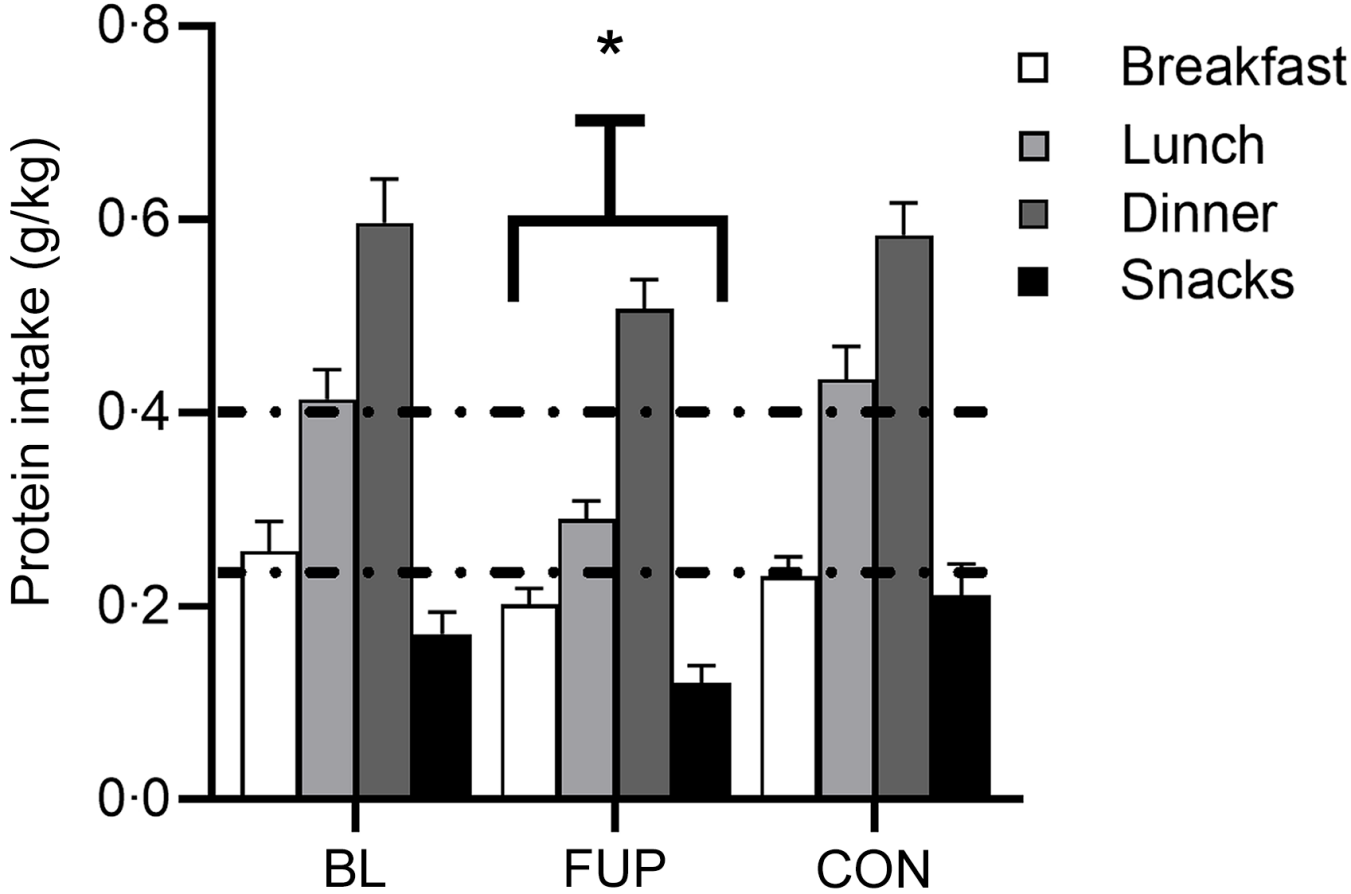
Meal-specific protein intake relative to bodyweight (in kilograms) for baseline (BL), follow-up (FUP) and healthy controls (CON) at breakfast (clear bars), lunch (light grey bars), dinner (dark grey bars) and as snacks (black bars). The dashed lines represent protein intake required for near maximal stimulation of muscle protein synthesis for younger (∼0·24 g·kg^–1^) and older (∼0·40 g·kg^–1^) individuals, respectively, taken from Moore *et al.* (2015)^(39)^. Values are presented as means (sd). Significance was set at *P* < 0·05. * Significantly different to BL and CON.

Meal-specific protein intakes are presented in Fig. 2 and Table 1. Daily dietary protein intake was distributed unevenly across meals with ∼18, 29, 41 and 12 % of protein in the CF group at BL and ∼15, 30, 42 and 13 % of protein in the CON group being consumed at breakfast, lunch, dinner and as snacks, respectively (Fig. 2). Similarly, in the CF group at FUP, protein intake remained unevenly distributed across meals (∼18, 26, 45, 11 % for breakfast, lunch, dinner and snacks, respectively).

Significant main effects for time and group were found for protein intake distribution for the CF group at FUP compared with the CON group and then for CF participants at FUP compared with BL (all *P* < 0·0001), but not between CF participants at BL compared with CON (all *P >* 0·65). However, no significant interaction effects were found (all *P* > 0·63). Whilst no differences were observed between CON group and CF at BL (BL: 0·41 (sd 0·19) g·kg^–1^, CON: 0·42 (sd 0·21) g·kg^–1^, *P* = 0·81, *d* = 0·05), Fig. 2), protein intake at lunch significantly reduced at FUP in CF participants compared to both CON and CF at BL (FUP: 0·29 (sd 0·12) g·kg^–1^, both *P* < 0·05, *d* > 0·76, Fig. 2). There was no statistically significant differences between CF at BL, CF at FUP and CON for protein intakes at breakfast (BL: 0·26 (sd 0·19) g·kg^–1^, FUP: 0·20 (sd 0·11) g·kg^–1^, CON: 0·21 (sd 0·13) g·kg^–1^, both *P* > 0·10, *d* < 0·35), dinner (BL: 0·60 (sd 0·29) g·kg^–1^, FUP: 0·51 (sd 0·19) g·kg^–1^, CON: 0·58 (sd 0·24) g·kg^–1^, both *P* > 0·08, *d* < 0·36) or with snacks (BL: 0·17 (sd 0·14) g·kg^–1^, FUP: 0·12 (sd 0·11) g·kg^–1^ and CON: 0·18 (sd 0·19) g·kg^–1^, both *P* > 0·07, *d* < 0·39). Across all groups, protein intakes were higher at dinner compared with breakfast and lunch and higher at lunch compared with breakfast (all *P* < 0·001).

On a meal-to-meal basis, the proposed dietary protein threshold for maximal MPS in younger individuals (0·24 g·kg^–1^) was only met on all recorded days by 10, 25 and 63 % of CF individuals at BL and 10, 15 and 58 % of CF individuals at FUP for breakfast, lunch and dinner, respectively. When expressed relative to the maximum threshold for older individuals (0·40 g·kg^–1^), the threshold was met on all 3 recorded days by 0, 8 and 33 % of CF individuals at BL and 0, 0 and 18 % of CF individuals at FUP for breakfast, lunch and dinner, respectively. Snacks were often not consumed as a single meal; therefore, this was not included analysis. There was no effect of sex, nor any differences in meal-specific protein intake between males and females (all *P* > 0·05, Table 1).

### Overall diet and protein quality

**Figure 3.**
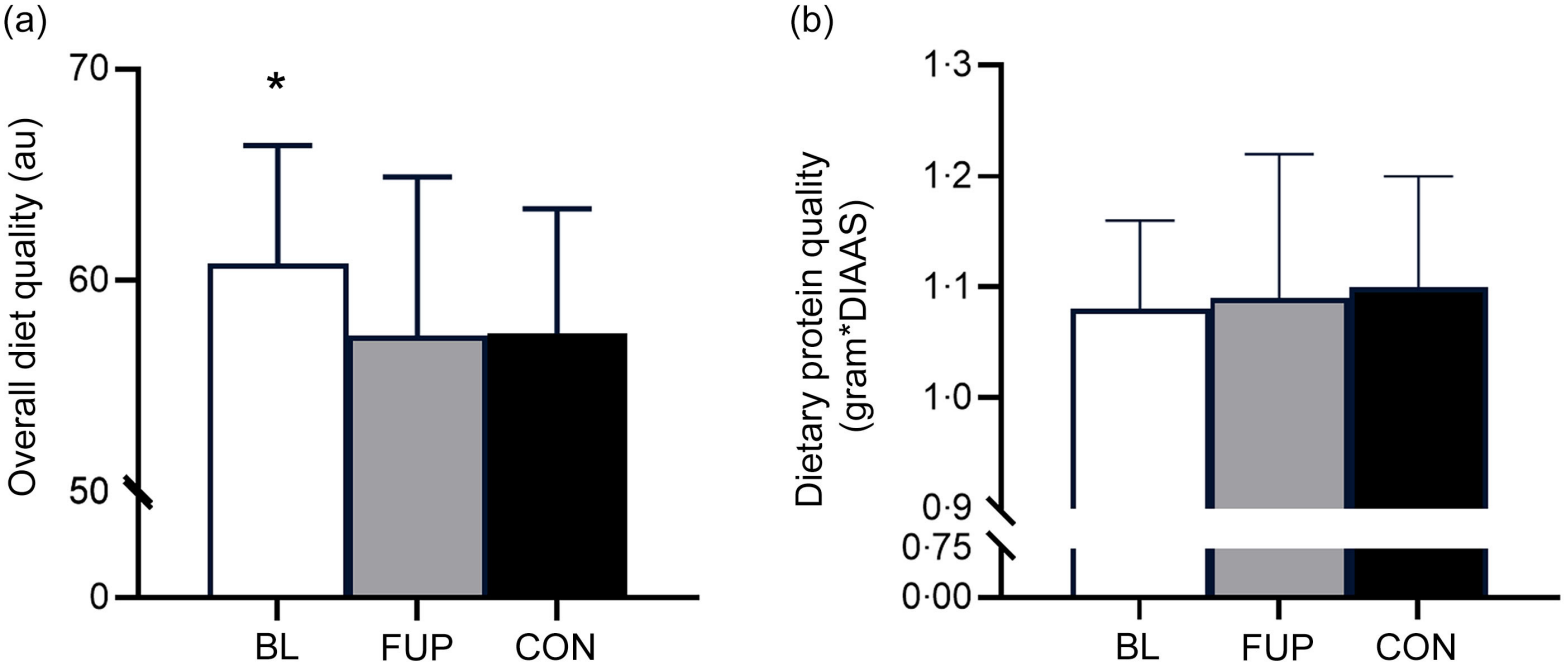
Overall diet quality and dietary protein quality for baseline (BL, clear bar), follow-up (FUP, grey bar) and healthy controls (CON, black bar) are shown in Panels A and B, respectively. Overall diet quality was assessed using the Healthy Eating Index (Panel A). Dietary protein quality was assessed by multiplying protein intake by the respective Digestible Indispensable Amino Acid Score (DIAAS), factoring in ileal digestibility for a single protein source (Panel B). Values are presented as means (sd). Significance was set at *P* < 0·05. * Significantly different to FUP and CON. *au*, arbitrary units.

Using Heating Eating Index as a marker of overall diet quality, it was found that diet quality was significantly higher in CF participants at BL compared with CON (BL: 60·8 (sd 5·6) *au*, CON: 57·4 (sd 7·5) *au (arbitrary units)*, *P* < 0·01, *d* = 0·51). However, diet quality was significantly reduced in the CF group at FUP compared with BL (FUP: 57·5 (sd 5·9) *au*, *P* = 0·027, *d* = 0·57). There was no difference in diet quality between CF participants at FUP and CON (Fig. 3(a), *P* = 0·79, *d* = 0·07). The most common source of protein intake across all groups was meat and poultry, with ∼76 %, ∼75 % and ∼75 % of protein intake of animal origin in CF at BL, CF at FUP and CON, respectively, consisting largely of meat and poultry (∼40–45 % across all groups), fish (∼4–5 % across all groups) and dairy/eggs (∼28–31 % across all groups). To facilitate a comparison of protein quality, we used a method for determining protein quality (the ‘Digestible Indispensable AA Score’) for a single source combined with the total consumption of each protein, as well as protein intake relative to total caloric intake. However, our analysis revealed no differences in protein quality between any groups using either marker of protein quality (all *P* > 0·05, Fig. 3(b)). There was no effect of sex, nor any differences in overall diet quality, protein quality or protein source between males and females (all *P* > 0·05).

## Discussion

To our knowledge, this is the first study comprehensively assessing dietary habits in awCF with an explicit focus on the amount, pattern, quality and source of dietary protein intake and overall diet quality, prior to and (> 3 months) following the commencement of ETI therapy, and compared with a healthy age-matched control group. We found that daily protein intake for awCF was higher than the RDA at both time points, with no differences between CON and CF at BL. However, in line with declines in total energy intake^(15)^, protein intake significantly reduced in CF at FUP, with a statistically significant effect on protein intake at lunch. Diet quality also significantly reduced at FUP in awCF compared with BL, to a level comparable to CON.

### Daily protein intake

Despite impaired nutrient absorption and reduced muscle mass and function in CF^(5,22,23,36)^, specific protein guidelines for pwCF are lacking, a notable omission noted within CF management guidelines^(44)^. This is perhaps unsurprising given the historic poor prognosis of CF and the use of a high-fat, high-calorie diet to maintain weight and minimise lung function decline and declines in overall health. The improvement in clinical stability following ETI therapy provides an opportunity for dietary modification with a focus on a healthy and balanced diet, including adequate protein to counteract CF and age-related complications such as the deteriorating skeletal muscle mass and function^(18)^.

Within the current study, the UK RDA for protein of 0·75 g·kg^−1^·day^−1^, which is based on the consumption of high-quality protein in order to satisfy daily EAA requirements, was met by the majority of CF and CON individuals. However, 70 % and 90 % of individuals with CF did not reach the higher recommended daily protein intake of 1·2 g·kg^−1^·day^−1^ on all measurement days at BL and FUP, respectively, compared with only 40 % in CON. These higher recommended levels have been proposed for older age^(39,40)^, as well respiratory diseases at risk of comorbid sarcopenia, such as COPD^(45)^, and are largely based on observations of attenuated muscle loss in those > 60 years old and on the notion of consuming 3 meals daily containing ∼0·40 g·kg^−1^ of protein, achieving a near maximal MPS response at each meal opportunity^(39,40)^. However, an uneven pattern of dietary protein intake was observed across meals for all groups, which was likely insufficient to reach the proposed threshold for maximal MPS stimulation at each meal and exacerbated by the lower total daily intake of protein in awCF at FUP.

The meal-specific distribution pattern of daily protein intake has been proposed as a factor in maximising stimulation of MPS across the day, with an evenly spread protein intake thought to enhance daily net postprandial muscle anabolism^(46)^. The majority of individuals in the present study failed to reach ‘*maximal’* MPS stimulation thresholds at all meal opportunities, particularly at breakfast, lunch and in participants on ETI therapy. In addition to a decline in overall food intake, this may in part reflect the requirement of consuming fat containing foods with ETI administration, which may have influenced breakfast or even lunch choice, if medication was delayed. The proposed dietary protein threshold for maximal MPS in young (0·24 g·kg^−1^) was only met on all recorded days by 10, 25 and 63 % of CF individuals at BL and 10, 15 and 58 % of CF individuals at FUP for breakfast, lunch and dinner, respectively. When expressed relative to the higher MPS threshold for older individuals (0·40 g·kg^−1^), the threshold was met on all recorded days each by 0, 8 and 33 % of CF individuals at BL and 0, 0 and 18 % of CF individuals at FUP for breakfast, lunch and dinner, respectively. Therefore, it is plausible that the dietary protein habits of our CF cohort are insufficient to support skeletal muscle mass, and even more so in older age, due to a failure to maximally, or even robustly, stimulate MPS with every meal. Importantly, even though we did not assess protein intake in an older CF population, (18–28 years old *n* 9; 29–39 years old *n* 18; 40–50 years old *n* 11; 51+ years *n* 2), yet still compared our data to protein thresholds for older age in CF, we would expect protein intake to continue to decline with increasing age, as has been demonstrated numerous times before^(37)^. Indeed, our data of the skewed distribution of protein intake are also consistent with observations in other cohorts of older individuals of varying health status^(37,47–49)^. However, our observations should be interpreted with caution as whilst data with isolated protein sources in an acute laboratory setting are encouraging, clear confirmatory data of the relative significance of this concept of per meal protein distribution across the day remained to be reported, particularly with whole-food studies which are more representative of habitual dietary patterns. It may, therefore, be more prudent based on current available evidence to focus on how many eating occasions an individual hits a proposed ‘*threshold*’, rather than the distribution of protein, *per se*.

### Protein and overall diet quality

The availability of sufficient EAAs within the diet is important for a robust increase in MPS and to support skeletal muscle remodelling^(29,50)^. Higher-quality proteins, reflected by superior digestible indispensable AA scores, have a greater protein density, greater EAA-to-Non-Essential AA ratio and a favourable EAA profile which closely matches the bodily needs^(29,50)^. Based on these characteristics, animal, rather than plant-based, proteins are generally considered to be higher quality^(33,34,48)^. This is particularly pertinent to note in a cohort such as pwCF who exhibit impaired nutrient absorption and higher nutritional needs, characteristics also observed with ageing^(51)^. Indeed, given their pre-existing nutrient absorption issues, consumption of a higher proportion of lower-quality plant-based proteins, which are less digestible and bioavailable^(33,34)^, might exacerbate nutrient status and malnutrition in CF. However, despite significant impairments in nutrient absorption and reduced muscle mass, quality, and function in pwCF to suggest a benefit for higher protein intakes for skeletal muscle regulation^(5,22,23,36)^, no guidelines exist for protein requirements in this population other than a general recommendation to increase food intake, perhaps owing in part to the poor historical prognosis of CF^(44)^. In the present study, no notable differences were observed between groups in protein sources consumed at each meal, nor protein quality (Fig. 3B). Nevertheless, substituting lower- for higher-quality proteins, particularly at lunch, may represent one viable easy-to-implement dietary approach to help to increase EAA delivery and support skeletal muscle maintenance in an ageing CF population. However, it is also worthy of note that breakfast is typically considered a particularly low protein dense meal and has been identified as an important opportunity to raise daily protein intake to combat age-related muscle deterioration^(46,52,53)^. This was supported by data presented herein across all groups (Fig. 2, Table 1), and therefore may benefit from the provision of an increase of higher quality proteins at this mealtime.

In contrast with our observations of no differences in dietary protein quality, overall diet quality was significantly higher in CF participants at BL, but this was significantly reduced at FUP to a level which was similar to our control group. Whilst a small change (5·5 % reduction in diet quality) over a short time period, this may represent a reduced focus on the quantity and quality of food consumption in pwCF on ETI therapy, potentially owing to the improved clinical stability and significant weight gain of pwCF, which was previously difficult to achieve^(15)^. By contrast, that diet quality was higher at BL in pwCF compared with CON may reflect an increased individual and clinical focus on the quality of food intake to help counteract significant digestive and nutrient absorption issues associated with CF pre-commencement of ETI therapy. A focus on diet quality may therefore be needed as a means to also to improve the quality of dietary protein and support metabolic and muscle health in pwCF.

### Experimental considerations and future directions

It is important to acknowledge several experimental considerations of and future directions for our work. First, a paucity of studies studying nutrient absorption and protein metabolism in pwCF make it difficult to formulate CF-specific MPS stimulatory thresholds and dietary protein recommendations^(15)^. Given the improvement in prognosis in pwCF, this represents an important avenue for future research. Our findings support calls for future studies to investigate first, whether anabolic resistance to protein provision is present in pwCF and subsequently whether increasing or redistributing per-meal protein intakes in CF, targeting breakfast and lunch, could maintain skeletal muscle health, particularly in an ageing CF population. Whether this is best achieved through protein supplementation, fortifying commonly consumed foods with protein/EAAs, or altering meal macronutrient composition in favour of protein, also remains to be elucidated. Further, given the high infection risk and inflammation observed in pwCF, which may also independently affect protein turnover^(54)^, assessing protein turnover at the whole-body level, combining the use of stable isotope tracers with non-invasive approaches (e.g. breath and urine to determine exogenous AA oxidation and retention, and 3-methylhistidine enrichments, respectively) may represent a particularly promising avenue for future work in CF.

In our study, it is important to acknowledge that we did not comprehensively assess the physical activity status or body composition changes of our CF participants. This is important to highlight as physical activity status may be an significant determinant of muscle anabolic responsiveness^(55)^. Specifically, physical activity/exercise act in synergy with dietary protein ingestion to further enhance MPS and can therefore improve muscle anabolic responsiveness in older individuals regularly failing to consume adequate daily protein amounts^(56–58)^. It is widely accepted that combining dietary protein strategies with regular physical activity, particularly in the form of structured resistance exercise training, offers the most potent non-pharmacological means of maintaining or improving muscle mass, strength and function in older age and represents an important consideration in an ageing CF population^(59–61)^. Knowledge of body composition changes in this study would also have been valuable, as a reduction in lean/muscle mass at FUP would have provided further strength to our suggestion of an increased consumption of dietary protein in CF to mitigate against muscle loss, and excess adiposity impairs metabolic health and can contribute to anabolic resistance to protein provision^(55,62)^. Whilst we acknowledge that our CF cohort is more representative of a younger-to-middle-aged population with specific reference to chronological age, we felt it was important to assess the implications for healthy ageing if protein intakes reduce at FUP and continue to decline thereafter, as observed in heathy adults^(37)^. In addition, as CF is associated with reduced muscle mass and function and a short life expectancy, this condition represents a model of accelerated ageing, thereby justifying the comparison with MPS thresholds for older adults, and declines in age-related muscle mass are actually generally apparent from middle-age onwards^(63)^.

It is prudent to acknowledge some of the valid limitations of the method of data collection primarily used for the data presented herein, including underreporting of food intake and over reporting of food quality (in accordance with social desirability) that may skew some of our observations^(64,65)^. However, high validity and precision have been reported for dietary records, particularly in clinical practice when used following adequate procedures and considering sufficient number of days^(64–66)^. In addition, diet diaries are regularly completed by this population as part of their clinical support programme, and the use of a well-trained facilitator was employed in the current study, thereby reducing some of the concerns typically associated with dietary records^(64–66)^. A notable limitation of our data though is the relatively short duration of ETI therapy (3 months), which may not be sufficient to capture long-term dietary or metabolic adaptations following ETI therapy in adults with CF and requires further investigation. Our data also lack supporting behavioural/qualitative data and future work should incorporate assessments of dietary preferences or eating behaviours to substantiate some of our interpretations. Similar to our control group, we also observed inherent variability across our CF participants, highlighting some of the limitations with interpretation of our data but that a personalised approach is likely essential when devising protein recommendations for pwCF, particularly as there may also be individuals where higher protein intakes are clinically contraindicated (e.g. significant renal impairment). Finally, given the anticipated improvements in life expectancy in CF as well as observations of weight gain, significant research in CF is warranted as we begin to observe an older, obesogenic, CF population for the first time. Indeed, ageing, obesity and periods of energy deficit are also associated with impaired protein turnover/anabolic resistance, a higher need for dietary protein and accelerated skeletal muscle deterioration^(55,62,67–69)^. Future work may wish to explore the consequences of changes in macronutrient distribution in CF, given that adiposity^(55)^, energy deficit (as a result of reducing adiposity)^(69)^ and essential fatty acid consumption (low levels of which are associated with energy restriction)^(70)^ are associated with alterations to protein turnover that may negatively affect muscle mass in pwCF.

### Conclusion

Daily protein intake was higher in CF participants than the current protein RDA of 0·75 g·kg^–1^·day^–1^. However, protein intake significantly reduced following initiation of ETI therapy. Meal-specific protein distribution was uneven and inadequate to repeatedly reach the proposed threshold for ‘*maximal’* MPS stimulation, with the majority of individuals not meeting the proposed higher protein recommendation of > 1·2 g·kg^–1^·day^–1^, which might be expected for a condition associated with severe suppurative lung disease, exaggerated innate inflammation, malabsorption and a recognised risk of sarcopenia and reduced muscle quality, even in younger pwCF. No differences in dietary protein quality or source were observed; however, diet quality was significantly reduced at follow-up in awCF, after commencing ETI therapy. Increasing the total intake and quality of dietary protein, particularly at breakfast and lunch, in combination with regular physical activity and exercise in CF could potentially help mitigate muscle loss to support an increasingly ageing CF population in. However, these changes need to form part of an overall more balanced diet to minimise longer-term age-related co-morbidities and complications.
